# Supplementary material for: Redesigning and validation of fertilizer use in maize for variable plant densities in central rift valley and Jimma in Ethiopia
Source: PLoS One. 2024 Jul 3;19(7):e0304004. doi: 10.1371/journal.pone.0304004 (PMC11221747; doi:10.1371/journal.pone.0304004)
Supplement: Attachment — Submitted filename: PONE-D-23-22722-Redesigning and Validation of Fertilizer Use in Maize for Variable Plant Densities and Farm Classes.pdf [file pone.0304004.s009.pdf]

**PONE-D-23-22722-Redesigning and Validation of Fertilizer Use in Maize for Variable Plant Densities and Farm Classes in Central Rift Valley and Jimma in Ethiopia**

Maize is a staple crop in many African counties and its availability is a measure of food security and any research leading to maize productivity leads to positive contribution. However, there are key fundamental concerns to be addressed in this study: From the title, the authors want to validate the fertilizer use- what necessitated the need for validation? At the end of the breeding trials, recommendations are usually made on plant density for each variety and it is not clear why the need for undertaking a trial on densities at this stage (no justification in the document). For fertilizers, this is understandable since most cases there are blanket recommendations without consideration of regions and soil fertility. Moreover, the word "redesigning" should be clearly defined as this sounds quite semantic.

**Abstract**

Generally, the abstract looks quite long, though this depends with Journal instructions. The abstract should be succinct, gives picture of the work and captures key aspects. I expected to see the general problem that necessitated this study. From this study, there is no gap that the authors aimed to fill and I was also unable to pick out the novelty of the work.

Keywords: the key words do not relate to the title of the manuscript.

Specifically, there is need to respond to following concerns:

Lines 11-12: "Redesigning and testing fertilizer use and plant densities in maize are important for grain self-sufficiency and livelihood improvement of smallholder farmers" –

* I do not seem to understand how redesigning and testing fertilizer use and plant densities play role in maize grain self-sufficiency. Moreover, there is need to define the term "redesigning". It may imply that there was "designing" already, and if that is the case, what is wrong with initial design to warrant the "redesigning"? Please make this come out.

The densities in (32,443 and 53,333 plants ha$^{-1}$ in Central Rift Valley (CRV); 27724 and 62,000 plants ha$^{-1}$ in Jimma):

*Why extreme comparisons in CRV? Why didn't the authors just adopt similar densities for both sites? Naturally, studies for densities recommend use of at least three so that it is possible observe yield plateau with increase in densities in order to make final conclusion on optimal density. Any comment on this?

Lines 14-15 "three farm classes (poor, medium and rich) in contrasting agro-ecologies of Ethiopia"-

*It is not clear whether the "farm classes" are part of experiment. Were these classes different in terms of soil nutrients levels?; Please clearly differentiate them.

**Introduction**

Lines 52-54 “Secondly, bringing more lands under cultivation is associated with increasing greenhouse gas emissions to the atmosphere which exacerbates climate change [6, 7]”-

*Can the authors explain how bringing more land under cultivation will exacerbate climate change. Otherwise can re-phrase the statement.

Lines 63-64 “The yield gap is the difference between the water-limited potential yield and the actual yield [9]”-

*Does it mean that yield gap can only be addressed through water management? What about use fertilizers and plant densities? Please comment on this.

Lines 67-70Accordingly, 12% more area (fields) were cultivated with maize in 2018 compared to the area occupied by the crop in 2014 whereas, the national average yield of maize increased from 3.4 t ha-1 in 2014 to 4 t $ha^{-1}$ in 2018 [10, 11] which is equivalent to 18 % additional yield over the past four (2014-2018) years.

--Please re-look at the statement; from the statement, the increase in maize production was due to increase in area cultivated and yet authors talk of 18%additional yield; yield addresses the production per unit area; or productivity which was not the case in this scenario.

85-86 “The study also unraveled whether farms different resource endowment need different crop management technologies”.

*The statement needs to be rephrased” at this state, use of the word “whether” is wanting.

Materials and Methods

Lines 96-99 “The minimum temperature ranges from 5°C to 16.8 °C in CRV whereas this temperature ranges from 5 °C to 17 in Jimma (Fig. 1). The maximum temperature in CRV and Jimma ranges from 18 °C to 31 °C and from 17°C to 32 °C respectively”

*Please re-look at the statements. The maximum or minimum temperature cannot be a range.

Lines 101-102 “Fig 1. Daily rainfall, minimum temperature and maximum temperature of CRV and Jimma in 2017 and 2018 years”

- The figure legends are missing and the caption not there. Make sure there are clear legends and the caption should be self- explanatory. The way it is currently, it conveys minimal information.

Lines 104-105 “Average fertilizer use and plant density of maize in the CRV and Jimma regions of Ethiopia are presented in Table 1”

- From table 1; there are rates of NPK as 0, 0, 0. Then current; 21.5, 12.8, 0.0 then redesigned : 40.8, 0.0 12.2 – These are very confusing since in one instance, K is omitted and the in the case of “redesigned scenario” it is the P that is omitted (for CRV) but for Jimma, the current is 53, 30, 0.0 and “redesigned” is 149.8, 9.0; 130.6…There is no clear explanation for this kind of adhoc applications. The omission/substation

experiment are always systematic and should have been for both sites. Surprisingly, in the table I did not see the farm class factors (poor, medium and rich): were this in terms of soil nutrient status?. And if economic status; then did this have bearing on amount of NPK supplied? If yes, then how do you handle the 3 levels of fertilizers and the interaction with farm factors?: In general, the treatments in this table should be explained carefully with justification for randomness of treatment. This kind of applications lead to numerous and complex interactions that cannot be interpreted and hence makes the whole research futile. Interestingly enough, the tables/figures presented were quite simple and do not wholly represent treatments presented in table 1.

Line 114: **2.3Nutrient amounts in redesigned fertilizer use**

- I guess this section is meant to provide methodology for part of objective 1:i.e to redesign and test fertilizer use and plant density under on-farm conditions and *unravel their effect on maize yield and yield gaps*-
- The authors are not clear on whether this was nutrient omission trial but they make mention of this, if so why do omission trial at this stage? However, based on table 1, if the omission trials were made, then other nutrient rates should have been held constant at every stage.
- Then there is also mention of yield gap issues – normally this is done through modelling but the questions is, why is it based on water-limitation and not nutrient? Why set it at 50%? And even in this instance, based on what plant density?
  -Generally, the whole contents of this section do not relate to the title of the manuscript.
- Lines 135-136 “In CRV, 136 12 farms (**4 poor, 4 medium and 4 rich**) were selected”- This statement is not clear at all. Does wealth status have influence on soil fertility or purchasing powers? And if so, to what extent?: Please have a table and show all variations in input availability and nutrient levels due to wealth status of farms- e.g. livestock, chicken etc. and show how this affected, say the nutrient status – Honestly, I do not understand why these aspects were brought up. They could have been part of a larger project but I do not think they are necessary in this manuscript.

Line 169 .2.6 internal nutrient use efficiency

It is not clear why this has popped up. It was not mentioned under “introduction section” and I seem not to relate it with any of the objectives. This section should clearly show the formulae computation of these efficiencies. There are several of these efficiencies, and it is not clear how the authors settled on what is presented in table 3.

**Line 215: Results**

There is no need of presenting ANOVA while presenting the findings (Table 2). Instead take this to supplementary section.

After Table 2, there are no line numbering hence difficult to write report.

Nevertheless, section 3.2 Yield responses to fertilizer use, plant density and farm class, the results presented in table 2 “Coefficient of variation (%) of maize yield in CRV and Jimma areas under various fertilizer uses and plant densities” do you think this adds any value to your work? Some of the CV values are as high as 60. How important is this? The CV interpretation depends on sensitivity of the work and authors’ experience with the work. I not see this coming out in the discussion section.

Note there was already another table 2 (please label appropriately).

In this table, abbreviations such as WOF, FFU, RDFU are not explained yet they are not universal. These should be clearly indicated in the table footnotes. Again, it seems the data were not analysed and hence not possible to tell if there were statistical differences between the treatments. The authors also make reference of figure 3. Unfortunately figures are not labelled. Without labelling the figures, it becomes difficult for readers/reviewers to understand the results.

Table 3- data not analysed. The authors provide maximum and minimum values instead of providing mean values. The means should then be compared amongst the treatments by having letter superscripts. These are lacking from this table

Please make sure all the figures are well labelled and captions indicated – the captions should be self- explanatory.

Due to the difficulties in interpreting the figures and lack of data analysis, the results need to clearly done. It is only then that discussions and subsequent sections can be meaningful. Hence I have not dedicated more time on reading the discussion.

Unless the result sections is seriously worked on, I am sorry so reject this work.
