# Supplementary material for: Redesigning and validation of fertilizer use in maize for variable plant densities in central rift valley and Jimma in Ethiopia
Source: PLoS One. 2024 Jul 3;19(7):e0304004. doi: 10.1371/journal.pone.0304004 (PMC11221747; doi:10.1371/journal.pone.0304004)
Supplement: Attachment — Submitted filename: Response_to_reviewers.docx [file pone.0304004.s010.docx]

24^th^ Dec, 2023

Subject: Response to PONE-D-23-22722 review

Dear editor and reviewers

We revised our manuscript and to the best of our understanding, concerns raised during the review were addressed. We believe that It is now

Kindly please accept our responses to the comments raised on the manuscript PONE-D-23-22722

**Reviewer #1**

**Comment**

Lines 11-12: “Redesigning and testing fertilizer use and plant densities in maize are important for grain self-sufficiency and livelihood improvement of smallholder farmers” – * I do not seem to understand how redesigning and testing fertilizer use and plant densities play role in maize grain self-sufficiency. Moreover, there is need to define the term “redesigning”. It may imply that there was “designing” already, and if that is the case, what is wrong with initial design to warrant the “redesigning”? Please make this come out.

**Response #1**

*Redesigning and testing fertilizer use and plant densities play role in maize grain self-sufficiency by enhancing adoption of the technologies which result in increasing grain productivity of the smallholder farmers. Farm survey showed sub-optimal fertilizer use and plant maize with low densities. Due to these reasons, we included ‘low adoption of the previous fertilizer use and plant density recommendations’ and to the best of our knowledge, such problem could be solved with validation approach and therefore the word ‘****redesigning****’ is seems appropriate as these technologies are already designed.*

**Comment**

The densities in (32,443 and 53,333 plants ha^-1^ in Central Rift Valley (CRV); 27724 and 62,000 plants ha^-1^ in Jimma):*Why extreme comparisons in CRV? Why didn’t the authors just adopt similar densities for both sites? Naturally, studies for densities recommend use of at least three so that it is possible observe yield plateau with increase in densities in order to make final conclusion on optimal density. Any comment on this?

**Response #2**

*The two sites are different in bio-physical potential (different agro-ecologies). CRV is characterized with sandy soil, low rainfall area and low maize potential (6.2 t/ha). However, Jimma is a humid region with loam soil, higher annual rainfall (up to 1500mm) and high maize yield potential (15 t/ha). So, different densities have been used in the regions and adapting similar densities in both regions seems illogical.*

**Comment**
It is not clear whether the “farm classes” are part of experiment. Were these classes different in terms of soil nutrients levels?; Please clearly differentiate them.

**Response #3**

*The farm classes were parts of the experiment and clearly indicated as factor levels on Table 1. The classes were based on resource endowments and we followed standard classification procedure such as cluster analysis using Nb clust package of R. The output was amended by local definition of farm class. As it becomes lengthy, we did not include this procedure in material and method. There was no large variation between farms in soil nutrients (see response #11). From resource use point of view, it is not advisable to conduct the validation experiment for each group of farms separately but with one run, it is possible to for all farms and reduces cost of the experimentation by 3. By using farms as factor, we get more information than not using the farm class as factor which enable to validate technologies and put in use for all farm types in the regions.*

**Comment**
Can the authors explain how bringing more land under cultivation will exacerbate climate change. Otherwise can re-phrase the statement.

**Response #4**

We accepted and rephrased the statement. We removed climate change and replace it with greenhouse gas emission.

**Comment**

Lines 63-64 “The yield gap is the difference between the water-limited potential yield and the actual yield [9]”- *Does it mean that yield gap can only be addressed through water management? What about use fertilizers and plant densities? Please comment on this.

**Response #5**

*The bench mark of the yield gaps are rainfall and irrigation. In this study, yield gap is the gap that exists under natural rainfall. In this case; it is addressed by* ***crop management practices*** *like fertilizer, plant density, panting date or tillage etc. But under irrigation system, it is addressed by both* ***water management*** *and* ***crop management****.*

**Comment**

Lines 67-70; Please re-look at the statement; from the statement, the increase in maize production was due to increase in area cultivated and yet authors talk of 18%additional yield; yield addresses the production per unit area; or productivity which was not the case in this scenario.

**Response #6:** *We accepted and revised to address this part.*

**Comment**

85-86 “The study also unraveled whether farms different resource endowment need different crop management technologies”. *The statement needs to be rephrased” at this state, use of the word “whether” is wanting.

**Response #7** *We accepted, rephrased as’…* *whether farms of different wealth status in CRV and Jimma require different fertilizer amounts and plant density for maize production’.*

Comments

*Please re-look at the statements. The maximum or minimum temperature cannot be a range. Lines 101-102 “Fig 1. Daily rainfall, minimum temperature and maximum temperature of CRV and Jimma in 2017 and 2018 years”
• The figure legends are missing and the caption not there. Make sure there are clear legends and the caption should be self- explanatory. The way it is currently, it conveys minimal information.

**Response #8**

*We accepted the comments and revised to address the concern of the reviewer (see the track change). The statement was rephrased (*Lines 96-99) *and clear fig replaced the former fig.*

**Comment (reviewer#1 and #2)**

From table 1; there are rates of NPK as 0, 0, 0. Then current; 21.5, 12.8, 0.0 then redesigned: 40.8, 0.0 12.2 – These are very confusing since in one instance, K is omitted and the in the case of “redesigned scenario” it is the P that is omitted (for CRV) but for Jimma, the current is 53, 30, 0.0 and “redesigned” is 149.8, 9.0; 130.6…There is no clear explanation for this kind of adhoc applications. The omission/substation experiments are always systematic and should have been for both sites. Surprisingly, in the table I did not see the farm class factors (poor, medium and rich): were this in terms of soil nutrient status?. And if economic status; then did this have bearing on amount of NPK supplied? If yes, then how do you handle the 3 levels of fertilizers and the interaction with farm factors?: In general, the treatments in this table should be explained carefully with justification for randomness of treatment. This kind of applications lead to numerous and complex interactions that cannot be interpreted and hence makes the whole research futile. Interestingly enough, the tables/figures presented were quite simple and do not wholly represent treatments presented in table 1.

**Responses #9**

We acknowledge the concerns of the reviewer. We have information of maize response to single nutrient (N, P or K) from NOT in Ethiopia (see Kenea et al., 2021; <https://doi.org/10.1016/j.eja.2020.126228>). In this study, we tried to deal with higher level complex and *tried to put it in practice. Let me make it more clear the goal of redesigning fertilizer use. The aim was to optimize NPK in terms of amount and proportion for a specific target yield. The target yield (50%*Yw) was selected because we can achieve this yield level under farmers’ management condition. The fertilizer treatments are different from nutrient omission trial and are explained as follow in CRV for example. In current (farmer’s use) (21.5:12.8:0 NPK) treatment, farmers are not using K and that is why K is 0. In redesigned use (40.8:0:12.2NPK), P was 0 kg/ha and it means that P supplied from soil was enough to achieve the target yield*. *The assumption was quantitatively estimated from the relationship of soil supply, target yield and physiological use efficiency of the crop using equation below.*

$$N_{i}\left( \frac{kg}{ha} \right)=\left( \frac{Y_{t}}{{PE}_{i}} \right)-{SS}_{i}$$

*Where Ni is N, P or K nutrient and Y_t_ is target yield; PE_i_ is the physiological use efficiency of the crop and SS_i_ is soil supplied N, P or K. On table 1, farm class is now included as factor.*

**Comment**

There is also mention of yield gap issues – normally this is done through modelling but the questions is, why is it based on water-limitation and not nutrient? Why set it at 50%? And even in this instance, based on what plant density?

**Response #10**

*The target yield (50%*Yw) was selected because we can achieve this yield level under farmers’ management condition. The modeling was based at recommended density (53,333 plants/ha in CRV and 44,444 plants/ha in Jimma. To the best of our knowledge, nutrient limited yield potential is not in use (not exist now). We know only two yield potentials: (1)* ***water limited potential yield*** *in which rainfall is a benchmark and (2)* ***potential yield*** *which is under optimal water (irrigation). In CRV where soil is poor and rainfall is not enough, 53,333plant/ha is believed to be enough and validation of this density enhances its adoption.*

**Comment**

Lines 135-136 “In CRV, 136 12 farms (4 poor, 4 medium and 4 rich) were selected”- This statement is not clear at all. Does wealth status have influence on soil fertility or purchasing powers? And if so, to what extent?: Please have a table and show all variations in input availability and nutrient levels due to wealth status of farms- e.g. livestock, chicken etc. and show how this affected, say the nutrient status – Honestly, I do not understand why these aspects were brought up. They could have been part of a larger project but I do not think they are necessary in this manuscript.

***Response #11***

*Soil fertility management strategy of farms can develop fertility gradient which most probably affect yield response of crops (See Zingore et al., 2007). The management strategy is mostly derived by wealth status of farms (purchasing power of farms, and cattle (manure availability). Despite the closeness of the values, variability of some soil fertility and tropical livestock unit (TLU) of farm categories are shown below.*

| Region | Farm class | Total N (g/kg) | OC (%) | Available P (ppm) | TLU |
| --- | --- | --- | --- | --- | --- |
| CRV | Rich | 1.38 | 1.57 | 10.64 | 10.6 |
|  | Medium | 1.25 | 1.35 | 5.67 | 10.3 |
|  | Poor | 1.19 | 1.27 | 8.00 | 6.80 |
| Jimma | Rich | 1.73 | 1.71 | 6.71 | 11.5 |
|  | medium | 1.50 | 1.16 | 6.63 | 6.4 |
|  | poor | 1.23 | 1.12 | 6.57 | 5.3 |

***Response #12 (internal use efficiency and ANOVA table)***

*Regarding the internal nutrient use efficiency, we accepted and introduced the terminology in the introduction. We also included reference in the materials and methods and did ANOVA in the result part. We also accepted and made revision to address the presentation of ANOVA table and presenting the findings. The ANOVA table is now moved to supplementary material.*

**Reviewer #2**

**Comment**

In the abstract (and perhaps in the result: “Increasing plant density from current to 53,333 plants ha^-1^ in CRV and to 62,000 plants ha^-1^ in Jimma led to an abrupt maize yield improvement in both regions.” Attributing the yield differences obtained due to difference in locations (that includes differences in soil, water, nutrient, varieties and many other factors) as a difference occurred due to plant density is not justifiable.

**Response #13**

*Interpretation was made per study area (per CRV and Jimma). The concern of the reviewer such as differences in soil, water, nutrient, varieties and many other factors exist between study area and constant within study area. To the best of our understanding, keeping these factors constant within regions, the interpretation ‘increasing plant density from current to redesigned resulted in increased maize yield is acceptable.*

**Comment**

In line 116-118, authors mentioned as “The target yield was set at 50% of Yw in CRV for both seasons. In Jimma, 50% of water-limited potential yield was used as target yield in 2017 whereas in 2018, 50 and 70% of Yw were used as target yields.” Where does this 70 % assumption comes from? Why it refer to only Jima area only for 2018? What is the justification for picking the target for redesigning fertilizer use at 50% of the water limited yield?

**Response #14**

*The 70% of Yw target yield was included by mistake in Jimma and removed. The justification of picking 50%Yw in redesigned fertilizer use was that it is possible to achieve 50% Yw at on farm, under farmer management condition. The redesigned amount (fertilizer) is indeed varies if the target yield varies.*

Comment

What is the basis for picking those plant densities as redesigned densities? How is this consideration of “redesigned density” different from the ordinary two level density experiment? i.e what is the redesigning concept introduced here?

***Response #15***

*The basis for picking of redesigned plant density (CRV =53333 plants ha^-1^ and 62,000 plants ha^-1^) is the previous demonstration trials and consult of senior agronomists. We also took soil resources and climatic elements (rainfall) into account. These densities believed to be the best in the respective region in terms of yield improvement and sustainable maize production.*

Comment

What is the justification for setting the current plant population as higher in CRV (32,443 plants ha^-1^) where moisture is the most limiting factor compared to Jimma area (27,724 plants ha^-1^) where much amount of seasonal rainfall is received and long maturing varieties are possible?

***Response #16***

*The justification of setting current density (CRV =32,443 plants ha-1 and 27,724 plants ha-1 is the farm survey output. These densities are the average density used by farmers in the respective result. Indeed, the current density in CRV is higher than the current density in Jimma that is the output of the study.*

Comments
Often mentioned as intermediate density. What does it mean and how does this relate to redesigning?
Line 145-146: The plot sizes in CRV and Jimma were 6 m × 6 m and 6.0 m × 6.4 m respectively. What is the reason for difference in size of plot between two locations? What is the rationale for classifying the farmers in to three categories when the treatments used are same i.e. the redesign of fertilizer use is based on targeting 50% of the water limited yield.

***Response #17***

*Intermediate density was another name for redesigned density. To address the concern and avoid confusion, we accepted the comment and changed to redesigned density. The reason for difference in plot size between the two locations was the difference in plant density in the locations. The rationale for classifying the farmers in to three categories is responded under response #3 and #11*

Comment

Coefficient of variability is not only generally high, but also mostly higher for the redesigned densities and in some cases (years) in redesigned fertilizers. What is the implication?

***Response #18****: Variability is high in redesigned densities*

**Comment**
Authors claim the following: Averaging on 2017 and 2018 cropping seasons, the achieved yield level by using redesigned fertilizer uses were 4.1 t ha^-1^ and 6.1 t ha^-1^ in CRV and Jimma, respectively, resulting in 65% and 38% of the water-limited potential yield of the crop in the respective regions.” How you end up with these percentages while you designed inputs to attain 50% of the water limited yield?

***Response #19***

*Indeed, the nutrients were redesigned to achieve the 50% of the water limited yield. In CRV however, we see an achievement of 65% of YW. This might be due to more conducive climatic condition (rainfall) than that was used for potential yield estimation or it might be due to the use of more perfect agronomic practices other than fertilizer. We do argue here as fertilizer is not the only yield determinant in crop production.*

**Comment**

The conclusion boldly claims: “The study investigated the importance of redesigning fertilizer use and plant density” whereas the result shows non-significant difference quit in a number of combinations”. How?

***Response #20****: We rephrased as ‘The study investigated the importance of fertilizer use and plant density’. The word redesigning is removed.*

**Reviewer #3**

L11-12: What is grain self-suffiency?

***Response #21***

*Grain self-suffiency is the ratio between supply and demand. In this study, it indicates the ratio of maize grain produced to the grain demand of the farm household.*

**L12: smallholder?**

***Response #22***

*Smallholder is mall scale farmer who manages land areas from less than 1-10 ha. In Ethiopia, they* *own 0.7 ha -2.7 ha land area. All agricultural activities mostly crop and livestock productions are conducted on this land.*

**Comment**
Organic carbon and N content of soil are closely related to the grain yield of maize compared to other soil and socioeconomic factors. - Is this information important? Why was it not in the abstract?

***Response #23***: *We accepted the comment and adapted in abstract, result and conclusion.*

Reviewer #4:

**Comment**
Line 119 – where did you get the information on nutrient content to compute physiological use efficiency?

***Response #24***

*We got the nutrient content from laboratory analysis for computing the physiological use efficiency. We computed uptake (kg/ha) and yield per uptake is a physiological use efficiency.*

**Comment**

Line 122 – how does the NOT relate to the trial sites? Can you expect the indigenous nutrient supply to be similar at the trial sites and the NOT?

**Response #25**

*The NOT sites in the same location with this study and soil supply was estimated from the previous NOT trial.*

**Comment**

Line 143/144: do you mean that one farm was one replicate?

Response #26: *Yes*

Line 146: what is the area that was used for harvest? Full plot? Net plot? Size of net plot? How was the time for harvest determined?

**Response #27**

*Harvesting was done from net plot size excluding the two border rows. The net plots were 6m ×4.8m in Jimma whereas in CRV the net plot size was 6m ×4.5m. Harvesting time was at physiological maturity when grain moisture was dropped to 15- 30% in most cases. This can be visually observed as leaves of the crop dries, cobs turns downwards from upright position.*

Comment
Line 150: where does the intermediate density come from? Before you were talking about redesigned density or optimum.

**Response #28**

*To be frank, intermediate density was included to mean the redesigned density and to avoid confusion; it is now changed to redesigned density.*

**Comment**

Line 156: what was the maturity duration of the varieties?

**Response #29**

*The maturity date of BH-660 (that was used in Jimma) was 160 days whereas BH-540 (that was used in CRV) was 140 days.*

**Comment**

Line 160: how old were the plants at the time of thinning?
**Response #30**

*The age of maize seedlings at thinning was 30 -45 days.*

**Comment**

What was the basis for the redesigned planting densities? You explain this for fertilizer, but not for the planting density.

***Responses#31***

*The bases of redesigning plant density are demonstration trial and consult of senior agronomists.*

**Comment**
Statistical analysis – this should be more comprehensive. You only explain how you compared treatments, but not how you established that there was a significant effect of a factor. Since you used several farms, did you use linear mixed modelling? Please provide the critical details. Did you perform statistical analysis for each region separately or in combination? Under results, you provide an ANOVA table that implies separate analysis. However figure 2 is only for Jimma – why did you omit CRV?

**Response #32**

The statistical analysis was conducted separately. From ANOVA table, we see that the interaction effect of fertilizer use and Density was significant only for Jimma which lead to produce fig 2result. Because, the interaction between fertilizer use and density was not significant for CRV, it was not presented.

**Comment**

Relation of maize yield to socio economic and soil factors. Am not an expert in the area, but nevertheless wondering if all the socio economic factors are meaningful here? Did you use any procedure to identify the best factors to be included in this analysis?

**Responses #33**

*Some factors were reduced and relevant factors were included under supplementary materials*.

**Comment**

Line 208: what is the difference between experimental and sampled yield?

***Response#34***

*Experimental yield is what is measured from experimental plot whereas sampled yield is what was measured from farmer’s field (average).*

**Comment**

Line 210/211: first you use an average yield of 2017 and 2018, then you say you used the sampled yield of 2017. Please explain.

**Response #35**

*For experimental yield, an average yield of on farm experiment in 2017 and 2018 was used, and for sampled yield, the yield sampled in 2017 from farmers’ fields was used.*
