# Supplementary material for: Redesigning and validation of fertilizer use in maize for variable plant densities in central rift valley and Jimma in Ethiopia
Source: PLoS One. 2024 Jul 3;19(7):e0304004. doi: 10.1371/journal.pone.0304004 (PMC11221747; doi:10.1371/journal.pone.0304004)
Supplement: Attachment — Submitted filename: Response to Reviewers.docx [file pone.0304004.s011.docx]

Dear editor and reviewers,

Thank you for your generous comments that improved our manuscript. We revised the manuscript based on the comments and major changes in this revision include:

1. Farm class is not considered as experimental factor. It is removed from all parts of the manuscript
2. Statistical analysis: it is re-conducted with linear mixed model using lme4 and LmerTest. All parts of the parts updated accordingly and interpreted accordingly.
3. Relation of maize yield with soil parameters: socioeconomic parameters and sampled yield are removed. Experimental yield is related with soil parameter (of the experimental fields).
4. Yield variability assessment based on CV category was removed.
5. Revision that were not included in the text during the previous revision are now included in the text.

Specific Reponses:

Reviewer #4: Reviewer #4:
The responses by the authors are mainly fine but I am surprised that they have not modified their text accordingly (and if I have overlooked that – it would help to indicate in the response where the adjustment was made (line number)

Comment
Line 119 – where did you get the information on nutrient content to compute physiological use efficiency?
Response #24
We got the nutrient content from laboratory analysis for computing the physiological use efficiency. We computed uptake (kg/ha) and yield per uptake is a physiological use efficiency.
ReRe: this has not been included into the text, also not from which trials samples were analysed and used here.

Response of the authors:

ReReRe: *Grain and straw samples were collected from the on-farm trial of the first season (2017) and the NPK content was analyzed at WUR. This nutrient concentration was used for physiological use efficiency. This text was included in the methodology line #160 in clean manuscript and at 197 line # in track change doc.*

Comment
Line 122 – how does the NOT relate to the trial sites? Can you expect the indigenous nutrient supply to be similar at the trial sites and the NOT?
Response #25
The NOT sites in the same location with this study and soil supply was estimated from the previous NOT trial.
ReRe: has the section with the NOTs been removed?

Response of the authors:

ReReRe: *This fertilizer use treatments are the estimated NPK for achieving the 50% yield potential of the crop. To make it clear and how it differs from NOT, explanation has been given under discussion with a title of methodological consideration. See line# 437-450 on the clean paper and 609-623 line # on the track change doc.*

Comment
Line 143/144: do you mean that one farm was one replicate?
Response #26: Yes
ReRe: not included in text

Response of the authors

ReReRe: *One farm was a replicate.* *Now it is included in the text at Line #127 on clean paper and line # 156-157 on track change.*

Line 146: what is the area that was used for harvest? Full plot? Net plot? Size of net plot? How was the time for harvest determined?
Response #27
Harvesting was done from net plot size excluding the two border rows. The net plots were 6m ×4.8m in Jimma whereas in CRV the net plot size was 6m ×4.5m. Harvesting time was at physiological maturity when grain moisture was dropped to 15- 30% in most cases. This can be visually observed as leaves of the crop dries, cobs turns downwards from upright position.
ReRe: not included in text

Response of the authors:

ReReRe: *These responses are now included in the text. The line numbers are 144-148 on the clean document and 174-178 line numbers on the track change.*

Comment
Line 150: where does the intermediate density come from? Before you were talking about redesigned density or optimum.
Response #28
To be frank, intermediate density was included to mean the redesigned density and to avoid confusion; it is now changed to redesigned density.
ReRe: OK

Comment
Line 156: what was the maturity duration of the varieties?
Response #29
The maturity date of BH-660 (that was used in Jimma) was 160 days whereas BH-540 (that was used in CRV) was 140 days.
ReRe: not included in text

Response:

ReReRe: *The maturity date of BH-660 (that was used in Jimma) was 160 days whereas BH-540 (that was used in CRV) was 140 days. The statement was included in the paper in line number 140-141 on clean paper and 170-171 line numbers on the track change doc.*


Comment
Line 160: how old were the plants at the time of thinning?
Response #30
The age of maize seedlings at thinning was 30 -45 days.
ReRe: not included in text

The age at which thinning was done was 30 -45 days after emergency and now included in the text. Line # 147-148.

Comment
What was the basis for the redesigned planting densities? You explain this for fertilizer, but not for the planting density.
Responses#31
The bases of redesigning plant density are demonstration trial and consult of senior agronomists.
ReRe: The response could be more elaborate and should have been included in the text

Response of the authors

ReReRe: *The basis of redesigned plant density (CRV=53,333 plants ha^-1^ and 62,000 plants ha^-1^) is the performances in previous demonstration trials and consultation of senior agronomists. Soil resources and climatic elements (rainfall) of the regions were taken into account. These are included in the text at line numbers 116-120 in clean paper and136-140 line numbers in tack change doc.*

Comment
Statistical analysis – this should be more comprehensive. You only explain how you compared treatments, but not how you established that there was a significant effect of a factor. Since you used several farms, did you use linear mixed modelling? Please provide the critical details. Did you perform statistical analysis for each region separately or in combination? Under results, you provide an ANOVA table that implies separate analysis. However figure 2 is only for Jimma – why did you omit CRV?
Response #32
The statistical analysis was conducted separately. From ANOVA table, we see that the interaction effect of fertilizer use and Density was significant only for Jimma which lead to produce fig 2result. Because, the interaction between fertilizer use and density was not significant for CRV, it was not presented.
ReRe: still not clear if mixed modelling was used or how else the multilocational situation was addressed.

Response of the authors:

ReReRe*: In the version, linear mixed model was used for the statistical analysis replacing the factorial anova in the previous version. The result and other parts of the paper were updated accordingly. This was included in 150-158 line numbers on the clean doc and180-188 line numbers in the track change.*

Comment
Relation of maize yield to socio economic and soil factors. Am not an expert in the area, but nevertheless wondering if all the socio economic factors are meaningful here? Did you use any procedure to identify the best factors to be included in this analysis?
Responses #33
Some factors were reduced and relevant factors were included under supplementary materials.
ReRe: A more comprehensive response would have been appreciated.

Response of the authors

*ReReRe:* *In this revised version, socio-economic parameters (TLU, N use, P use, cropped area, maize area) and sampled yield are removed. Only the relation of experimental yield and soil parameter (of the experimental fields) were assessed. See 196-201 line numbers on clean paper.*

Comment
Line 208: what is the difference between experimental and sampled yield?
Response#34
Experimental yield is what is measured from experimental plot whereas sampled yield is what was measured from farmer’s field (average).
ReRe: still not clear from the text

Response of the authors,

ReReRe: *In this version, only one maize yield exists, the experimental yield. Sampled yield is removed (see the above response).*

Comment
Line 210/211: first you use an average yield of 2017 and 2018, then you say you used the sampled yield of 2017. Please explain.
Response #35
For experimental yield, an average yield of on farm experiment in 2017 and 2018 was used, and for sampled yield, the yield sampled in 2017 from farmers’ fields was used.
ReRe: OK, if above is clarified in the text

Response of the authors:

ReReRe*: the experimental yield that was related with the soil factors is the average yield of 2017and 2018 season grown under NPK (redesigned fertilizer use). Under NPK (redesigned fertilizer use) we assume that there was no yield limitation from nutrient perspective. Now there is no sampled yield in the paper and it might be clear. See line 334-340 of the paper (clean paper) and 468-488on track change. See also fig 4.*
